# STAT4 targets *KISS1* to promote the apoptosis of ovarian granulosa cells

**DOI:** 10.1186/s13048-020-00741-5

**Published:** 2020-11-20

**Authors:** Yao Jiang, Xiaoping Xin, Xiangchun Pan, Ailing Zhang, Zhe Zhang, Jiaqi Li, Xiaolong Yuan

**Affiliations:** 1 Guangdong Provincial Key Lab of Agro-Animal Genomics and Molecular Breeding, National Engineering Research Centre for Breeding Swine Industry, College of Animal Science, South China Agricultural University, Guangzhou, Guangdong China; 2 Guangdong Provincial Key Laboratory of Laboratory Animals, Guangdong Laboratory Animals Monitoring Institute, Guangzhou, Guangdong China; 3 College of Biology and Food Engineering/Development, Center of Applied Ecology and Ecological Engineering in Universities, Guangdong University of Education, Guangzhou, 510303 China

**Keywords:** *STAT4*, *KISS1*, Cell apoptosis, Synthesis of E2, Ovarian granulosa cells

## Abstract

**Background:**

In mammals, it is known that the estradiol-17β (E2) is mainly synthetized in ovarian granulosa cells (GCs), and the excessive apoptosis of GCs induces the follicular atresia. Many studies have implicated the essential role of *KISS1,* with the pro-synthetic effect of E2 and the anti-apoptotic effect on GCs, in the mammalian folliculogenesis, and several STAT4 potential binding sites were previously predicted on the promoter of *KISS1* in pigs. However, the biological effects of *STAT4* on GCs and the molecular regulation between *STAT4* and *KISS1* remained largely unknown.

**Methods:**

Using the porcine GCs as the cellular model, the overexpression plasmid, small interfering RNA, 5′-deletion and luciferase assay were applied to investigate the molecular mechanisms for STAT4 regulating the expression of *KISS1*.

**Results:**

In this study, the STAT4 negatively regulated the mRNA and protein levels of *KISS1* in porcine GCs, and the mRNA level of *STAT4* was observed to significantly decrease from immature to mature follicles, which was inversed with that of *KISS1*. The relative luciferase activity of *KISS1* promoter was significantly increased with deletion of the fourth potential binding site (− 305/− 295), and ChIP further confirmed that the STAT4 bound at − 305/− 295 region of *KISS1*. Besides, the STAT4 significantly regulated the mRNA levels of *PDK1, FOXO3* and *TSC2* of PI3K signaling pathway to promote the cell apoptosis and the percentage of cells at G0/G1 phase of cell cycle in GCs. Alternatively, the STAT4 significantly decreased the mRNA levels of *CYP17*, *3B-HSD*, *17B-*33 *HSD, ESR1,* and *ESR2*, as well as the concentration of E2 in GCs. Furthermore, interfering with the expression of *STAT4* was observed to significantly stimulate the pro-synthetic effect of E2 and anti-apoptotic effect of *KISS1* in GCs.

**Conclusions:**

Collectively, the STAT4 might directly target at − 305/− 295 region of *KISS1* to negatively regulate the transcription of *KISS1*, promote the cell apoptosis via PI3K signaling pathway, suppress the synthesis of E2 through the estrogen signaling pathway in porcine GCs. These proposed works could provide useful insight in further investigations on the molecular functionalities of *STAT4* and *KISS1* in the folliculogenesis of mammals.

## Background

In mammals, the Kisspeptin encoded by Kiss-1 metastasis-suppressor (*KISS1*) gene regulates the release of gonadotropin releasing hormone at the hypothalamic level [1, 2] and is widely known for its essential role in controlling of the initiating of puberty [3, 4]. Recently, the Kisspeptin has been reported to show the local roles in the ovaries of humans [5], pigs [6, 7], rats [8], hamsters [9], dogs [10], and cats [11]. It is observed that the *KISS1*^−/−^ mice fail to undergo pubertal transition and show the absence of mature follicles [12, 13]. In the ovaries of rats [8] and dogs [10], the mRNA of *KISS1* expresses in a distinctive stage-specific pattern throughout the estrous cycle. In cats, the mRNA level of *KISS1* in follicular stage expresses higher than in luteal stage [11]. In pigs, the mRNA level of *KISS1* is found to significantly increase from immature to mature follicles [6]. These observations highly implicate the essential role of *KISS1* in the mammalian folliculogenesis.

Previous studies have suggested that the signal transducer and activator of transcription (STAT) family of cytosolic transcription factors [14, 15] appears to be phosphorylated to regulate the apoptosis and differentiation of various cells [16]. Many studies have reported that the STAT proteins highly express in granulosa cells (GCs) [17, 18] and play an essential role in folliculogenesis [19] and female reproduction [17, 20]. The knockout of GC-specific STAT3 significantly reduces the litter sizes of mice [20]. The levels of phosphorylated STAT3 in GCs of subordinate follicles are markedly higher than dominant follicles in bovines [21, 22]. More recently, the STAT4 has been linked to the ovarian endometriosis in women [23]. These results indicate that STATs may play an important role in mammalian folliculogenesis.

In mammals, it is known that estradiol-17β (E2) is predominantly synthetized in GCs, and the apoptosis of GCs plays an important role in deciding the fate of follicles [24–26]. The abnormal apoptosis of GCs induces follicular atresia [27] and exhibits a negative influence on oocyte quality [28] and fertility [29, 30]. The phosphatidylinositol 3-OH-kinase (PI3K) signaling pathway has been reported to promote cell survival and suppress apoptosis in mammalian GCs [31, 32] and is crucial for follicle growth [33]. Previously, we have found that the *KISS1* suppresses the apoptosis and cycle entry through PI3K signaling pathway in GCs to promote the maturation of follicles [6]. Besides, the mice deficient in *STAT4* demonstrate that *STAT4* induces the expression of genes involved in the proliferation and apoptosis of diverse cells [34, 35]. Moreover, several STAT4 putative binding locations were predicted on the promoter of *KISS1* in pigs. Therefore, we hypothesized that *STAT4* might bind at the promoter of *KISS1*, regulate the cell apoptosis and E2 secretion in GCs, and then promote the follicular development.

In this study, the molecular mechanism for the regulation between *STAT4* and *KISS1* was first determined, and then their biological functions were explored on PI3K signaling pathway, cell cycle entry, cell apoptosis, estrogen signaling pathway, and E2 secretion in porcine GCs. These works will provide useful information on the molecular mechanism of *STAT4*-mediated-*KISS1* in mammalian folliculogenesis.

## Materials and methods

### Ethics approval

All experiments in the present study were performed in accordance with the guidelines of the Animal Care and Use Committee of South China Agricultural University Guangzhou, China (Approval Number: SCAU#2013–10).

### Animals and sample preparation

The details of animals and sample preparations were previously described in our study [6]. Briefly, three Landrace × Yorkshire crossbred gilts with the exhibitions of the first estrus were selected [36, 37]. At least three of follicles within 8–10 mm and 5–7 mm were respectively collected and were respectively considered as the mature and immature follicles. These pigs were reared in the same conditions and fed the same diet daily. The collected follicles were frozen in liquid nitrogen and then transferred to store at − 80 °C fridge for further using.

### Prediction of potential STAT4 binding sites at the promoter of KISS1

The promoter sequences of porcine *KISS1* (upstream 2.5 kb) were download and acquired from NCBI (https://www.ncbi.nlm.nih.gov/gene/100145896). TFBIND [38], Biobase (http://gene-regulation.com/pub/programs/alibaba2/index.html), Jaspar [39] and Research (http://alggen.lsi.upc.es/cgi-bin/promo_v3/promo/promoinit.cgi?dirDB=TF_8.3) were applied to predict the putative and potential binding site of STAT4. The putative binding sites of STAT4 predicted by all of those four tools were selected for further using. The locations of these potential binding sites on the promoter of *KISS* are shown in Fig. 2a.

### Construction of KISS1 5′ deletion and luciferase assay

The total genomic DNA of porcine ovaries was extracted and used as the template. PCR was conducted by using PrimerSTAR® (TaKaRa, Dalian, Liaoning, China) enzyme to obtain the promoter sequences of *KISS1* (2482 bp). Then PCR products were purified to combine with pMD-18 T and transformed into competent cells DH5α. These DH5α were inoculated on ampicillin-containing lysogeny broth plates at 37 °C for overnight, and then the monoclonal bacteria was selected and amplified overnight at 37 °C shaker. The plasmids of bacteria were collected and extracted. The correct plasmid after sequencing identification was named T-*KISS1*. Then T-*KISS1 was used* as a template to design other five deletion fragments. The same method was used to acquire plasmids of each deletion fragment including P0 (− 2261/+ 221), P1 (− 1985/+ 221), P2 (− 1574/+ 221), P3 (− 1161/+ 221), P4 (− 850/+ 221), and P5 (− 289/+ 221) with *Sac*I and *Sma*I cleavage sites (Table 1). Finally, each deletion fragment was cloned into pGL3-Basic. According to Promega’s dual luciferase reporter assay kit (Promega, Madison, WI, USA) and previous study [40], the BioTek Synergy 2 multifunctional microplate reader (BioTek, Winooski, VT, USA) was used for fluorescence detection. The relative expression of firefly luciferase to renilla luciferase was targeted as the fragment activity. The primers used in this study are presented in Table 1.

**Table 1:** Primers for Construction of KISS1 5′ Deletion

Name	Sequence	Product (bp)	Accession number
P0(−2261/+ 221)	F: CGCGAGCTCCCTCACTCACCAGCCTGTTTC	2482	NM_001134964.1
	R: CCCCCGGGGGCCAGCCAGTCTTAGGTTTCCATTA		
P1(−1985/+ 221)	F: CGCGAGCTCGACATCCCTCACTCCCTACTACCC	2206	NM_001134964.1
	ditto		
P2(−1574/+ 221)	F: CGCGAGCTCTGGGTTTCAGGGTATCACAGAGC	1795	NM_001134964.1
	ditto		
P3(−1161/+ 221)	F: CGCGAGCTCGGCTCGCCAGTGGTTTATCTTT	1382	NM_001134964.1
	ditto		
P4(−850/+ 221)	F: CGCGAGCTCTAAGGGTTATGAGAGCAAGCAGGAT	1071	NM_001134964.1
	ditto		
P5(−289/+ 221)	F: CGCGAGCTCTGCTATTCAGACTCATCCCTCCACT	510	NM_001134964.1
	ditto		

* The underlined is enzyme-cutting sites

### Culture of porcine GCs in vitro

The porcine ovarian GCs were cultured as previously described [40, 41]. Briefly, the ovaries, which were collected from a local slaughterhouse (Guangzhou) for pigs, were transferred to laboratory in PBS (Invitrogen, Shanghai, China). Subsequently, the GCs were collected and isolated from 5 to 7 mm follicles, and then these GCs were washed twice with PBS. The GCs were cultured and seeded into 25-cm^2^ flasks within 37 °C and 5% CO2 in DMEM (Hyclone, Logan, UT, USA) containing 10% fetal bovine serum (Hyclone), 100 IU/mL penicillin, and 100 μg/mL streptomycin.

### Real - time quantitative PCR analysis

The pcDNA3.1-Basic (200 ng), pcDNA3.1-STAT4 (200 ng), pcDNA3.1-KISS1 (200 ng), siRNA-STAT4 (50 nM), or siRNA-NC (50 nM) was transfected into the cells for 48 h respectively, while GCs covered 30–50% of one well. The total RNA was collected and extracted for at least triplicates per group by using TRIzol reagent (TaKaRa, Tokyo, Japan) and then reverse-transcribed using a RevertAid First Strand cDNA Synthesis Kit (Thermo Scientific, USA) for mRNAs. The Maxima SYBR Green qRT-PCR Master Mix (2×) (Thermo Scientific) was used to quantify the relative expression levels of mRNAs in a LightCycler Real-Time PCR system. The relative mRNA expression levels of genes were calculated using the 2^-ΔΔct^ strategy with *GAPDH* as the endogenous controls. The primer sequences are listed in Table 2.

**Table 2:** Primers of RT-PCR, ChIP and coding sequences cloning

Name	Sequence	Product (bp)	Accession number
CDS-STAT4	F: GGGGTACCATGTCTCAGTGGAATCAAGTC	2266	NM_001197305.1
	R: CCAAGCTTTCAGTCTGAGTCAGGTCCTT		
CDS-KISS1	F: CCGAATTCATGAATGCACTGGTTTTTTGG	431	NM_001134964.1
	R: GGCGCCGGCGAGTCAGAGCGGGCCGCGGAA		
qRT-PCR-KISS1	F: AACCAGCATCTTCTCACCAGG	192	NM_001134964.1
	R: CTTTCTCTCCGCACAACGC		
qRT-PCR-STAT4	F: TTGTCTGCTCTACCATTCGCTG	182	NM_001197305.1
	R: TAACCTTTGTCTCCCCTTTCTG		
qRT-PCR-GAPDH	F: TCCCGCCAACATCAAAT	163	XM_021091114.1
	R: CACGCCCATCACAAACAT		
qRT-PCR-PIK3CG	F: AACGGGCTTTGAGATAGTGAA	184	NM_213939.1
	R: AAGTTGCTTGGTTGGTGGATA		
qRT-PCR-PIK3C1	F: CAAGTGAGAATGGTCCGAATG	152	NM_006218.3
	R: GTGGAAGAGTTTGCCTGTTTT		
qRT-PCR-PDK1	F: AAATCACCAGGACAGCCAATA	190	NM_001159608.1
	R: CTTCTCGGTCACTCATCTTCAC		
qRT-PCR-FOXO3	F: ACAAACGGCTCACTCTGTCCCA	85	NM_001135959.1
	R: GAACTGTTGCTGTCGCCCTTATC		
qRT-PCR-TSC2	F: CGAGGTGGTGTCCTACGAGAT	115	XM_005655162.3
	R: GAGCAGGCGTTCAATGATGTT		
qRT-PCR-BAD	F: AGTCGCCACTGCTCTTACCC	172	XM_021082883.1
	R: TCTTGAAGGAACCCTGGAAATC		
qRT-PCR-Star	F: GGAAAAGACACAGTCATCACCCAT	121	NM_213755.2
	R: CAGCAAGCACACACACGGAAC		
qRT-PCR-CYP17	F: AAGCCAAGACGAACGCAGAAAG	228	NM_214428.1
	R: TAGATGGGGCACGATTGAAACC		
qRT-PCR-3B-HSD	F: GGGGCTTCTGTCTTGATTCCA	284	NM_001004049.2
	R: GGTTTTCAGTGCTTCCTTGTGC		
qRT-PCR-17B-HSD	F: CCCAACGCAGGAGACTCAAAAT	149	NM_214306.1
	R: CCAGAGCCCATAACGAAGACAGA		
qRT-PCR-CYP19A	F: GCTGGACACCTCTAACAACCTCTT	91	NM_214430.1
	R: TTGCCATTCATCAAAATAACCCT		
qRT-PCR-ESR1	F: GATGCCTTGGTCTGGGTGAT	124	XM_003468423.2
	R: AGTGTTCCGTGCCCTTGTTA		
qRT-PCR-ESR2	F: AAGGGAAAAGGAGGATGGGACA	202	NM_0010011533.1
	R: CAGATAGGGACTGCGTGGAGGT		
ChIP-STAT4	F: CCTTGCCCACTTCACTCCAC	153	NM_001197305.1
	R: AGGACAGAAGGAATCGAGGGA		
ChIP-GAPDH	F: GATGTCCTGAGCCCCTACAG	102	XM_021091114.1
	R: GGTAGGTGATGGGGACTGAG		

* The underlined is enzyme-cutting sites

### Cell apoptosis assay

The coding sequences of *STAT4* (Gene ID: 397261, accession number: NM_001197305.1) and *KISS1* (Gene ID: 100145896, accession Number: NM_001134964.1) were cloned into pcDNA3.1(+) (ThermoFisher, Guangzhou, China) with the restrictive enzymes of *Kpn*I and *xho*I for *STAT4*; *EcoR*I and *Not*I for *KISS1*. The sequences of the primers for these coding sequences were shown in Table 2. STAT4-siRNA-1, STAT4-siRNA-2, STAT4-siRNA-3 and Scrambled-siRNA were synthesized and purified by RiboBio Co. Ltd. (Guangzhou, China).

According to our previous studies [40, 41], the cell apoptosis was detected to by using an Annexin V-FITC Apoptosis Detection Kit (BioVision, Milpitas, CA, USA). Briefly, when GCs covered 30–50% of the triplicate in 6-well plates at 24 h prior to transfection, pcDNA3.1-STAT4, pcDNA3.1-KISS1, pcDNA3.1-Basic, STAT4-siRNA, and Scrambled-siRNA were transfected into the cells for 48 h, respectively. Then the transfected cells were harvested and treated by using Annexin V-FITC mix and were analyzed in a flow cytometer (Becton Dickinson Co., San Jose, CA, USA). All experiments were performed at least triplicate.

### Cell cycle analysis

The analysis of cell cycle was according to our previous studies [40, 41]. When GCs covered 30–50% of one well, pcDNA3.1-STAT4, pcDNA3.1-Basic, STAT4-siRNA, or Scrambled-siRNA was transfected into GCs for 48 h, respectively. The transfected cells were collected and washed twice with ice-cold PBS. These cells were resuspended using a propidium iodide/RNase A solution at 37 °C for 30 min in the dark. Then these cells were analyzed by flow cytometry (Becton Dickinson Co., San Jose, CA, USA).

### ELISA for measurements of steroid hormones

After transduction with pcDNA3.1-STAT4, pcDNA3.1-KISS1, pcDNA3.1-Basic, STAT4-siRNA, and Scrambled-siRNA for 48 h, the concentrations of E2 in the culture supernatants were measured with ELISA kits (Beijing north institute of biological technology, Beijing, China) according to our previous studies [40, 41].

### Data analysis

Data were expressed as means ± standard deviation (SD) of repeated experiments. The student’s t-test (two-tailed) was used to detect the significance of mean differences between two groups by using R software (version-3.4.3, https://www.r-project.org/) in this study. * indicates *P* < 0.05; ** indicates *P* < 0.01.

## Results

### STAT4 inhibits the mRNA and protein expressions of KISS1 in porcine GCs

**Fig. 1:**
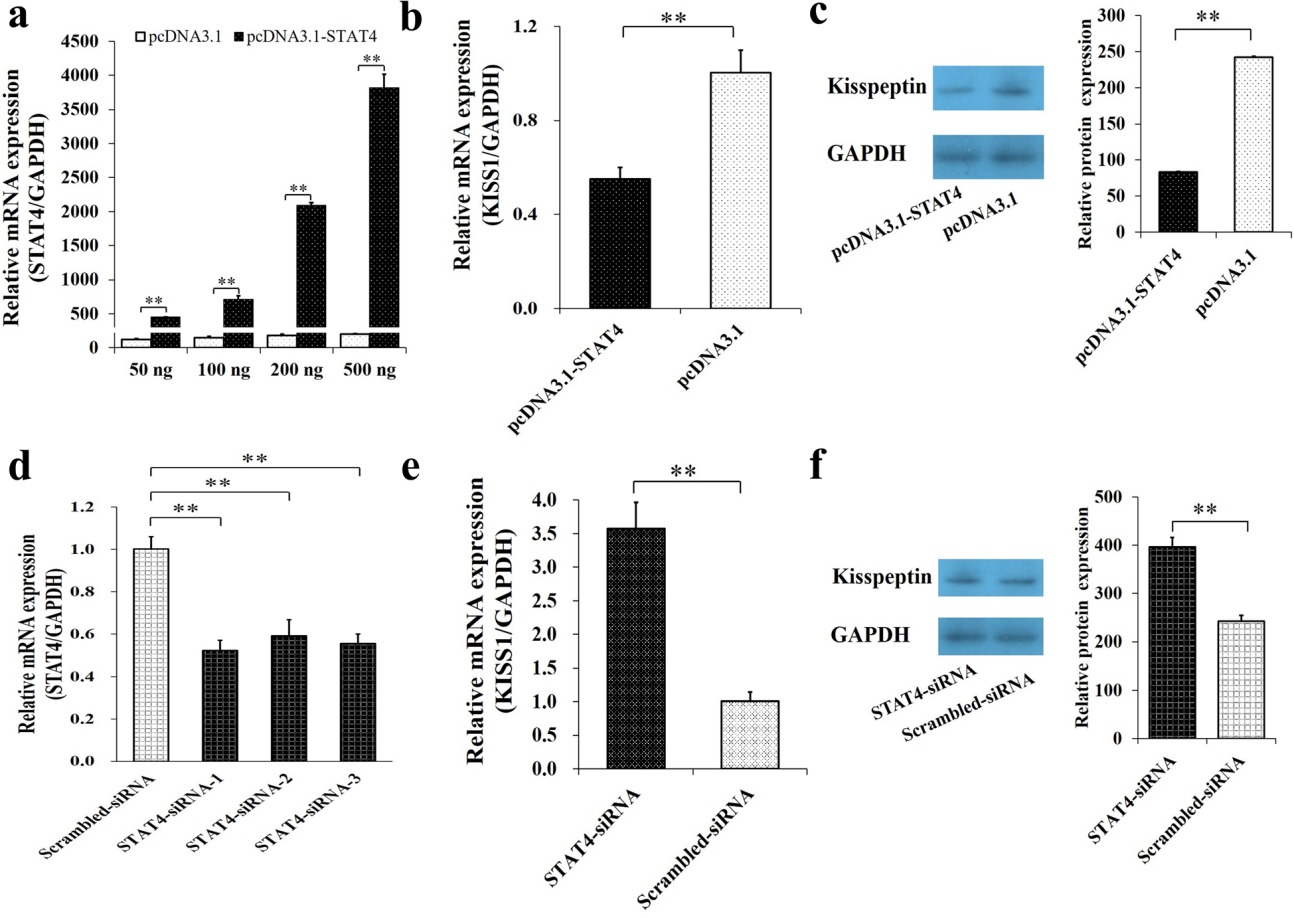
*STAT4* inhibits the mRNA and protein levels of *KISS1*. **a** Relative expression of *STAT4* against the different concentrations of pcDNA3.1-STAT4 plasmid; The mRNA (**b**) and protein (**c**) expressions of *KISS1* were depressed by pcDNA3.1-STAT4; **d** Relative mRNA expression of *STAT4* knockdown by three siRNAs; The mRNA (**e**) and protein (**f**) expressions of *STAT4* were stimulated by STAT4-siRNA. ** indicates *P* < 0.01. Data were represented as means ± SD. siRNA: small interfering RNA; Scrambled-siRNA: a siRNA negative control

The mRNA level of *STAT4* was found to be increasing with the concentration of overexpression plasmid (pcDNA3.1-STAT4) for *STAT4* (Fig. 1a), and 200 ng plasmid of pcDNA3.1-STAT4 was selected for further using on the considerations for cellular tolerance. The overexpression of *STAT4* significantly downregulated the mRNA (Fig. 1b, *P* < 0.01) and protein levels (Fig. 1c, *P* < 0.01) of *KISS1*, compared to the control group. Besides, three STAT4-specific small interfering RNA (siRNA) and a negative control (Scrambled-siRNA) were transfected into GCs to evaluate the knockdown efficiency for *STAT4* (Fig. 1d), and STAT4-siRNA2 was observed to show the highest performance and then was selected to inhibit the expression of *STAT4* in GCs (Fig. 1d). We found that interfering with the expression of *STAT4* significantly increased the mRNA (Fig. 1e, *P* < 0.01) and protein levels (Fig. 1f, *P* < 0.01) of *KISS1*, compared to the control group. These results indicated that *STAT4* negatively regulated the mRNA and protein expressions of *KISS1* in porcine GCs.

### STAT4 binds at − 305/− 295 region of KISS1

**Fig. 2:**
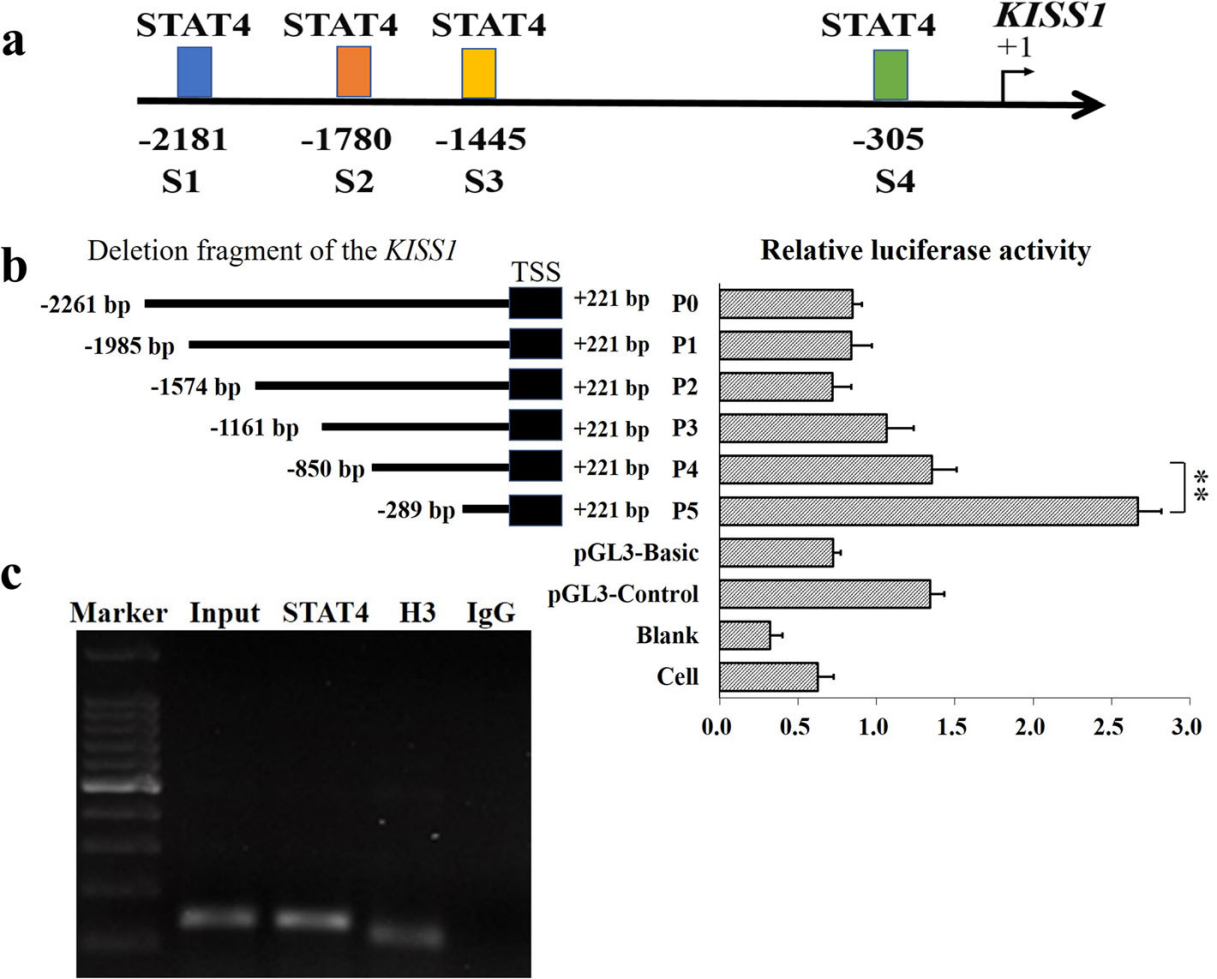
*STAT4* binds at − 305/− 295 region of *KISS1*. **a** Predictions of the potential binding sites of STAT4 at the promoter of *KISS1* in pigs; **b** Relative luciferase activity of *KISS1* promoter after 5′ deletion of the potential binding sites of *STAT4*; **c** Confirmation of STAT4 binding at − 305/− 295 by ChIP. ** indicates *P* < 0.01. Data were represented as means ± SD

Four potential binding sites of STAT4 were found at the promoter of *KISS1* (Fig. 2a), indicating that the STAT4 might directly target at *KISS1* to regulate the expression of *KISS1*. To study the molecular regulation and mechanism between *STAT4* and *KISS1*, the 5′-deletion reporter was built and constructed for *KISS1* (Fig. 2b). The deletion of S1 (− 2181/− 2171), S2 (− 1780/− 1770), and S3 (− 1445/− 1435) did not show significant changes on the relative luciferase activity of P1, P2, and P3, compared to P0 (Fig. 2b). However, compared to P0, P1, P2, P3, and P4, the deletion of the fourth potential binding site (− 305/− 295) significantly increased the relative luciferase activity (P5) (Fig. 2b). Moreover, ChIP further identified that *STAT4* bound at − 305/− 295 in porcine GCs (Fig. 2c). These results demonstrated that *STAT4* directly bound at − 305/− 295 region of *KISS1* to negatively regulate the transcription of *KISS1* in porcine GCs.

### STAT4 promotes the apoptosis of porcine GCs

**Fig. 3:**
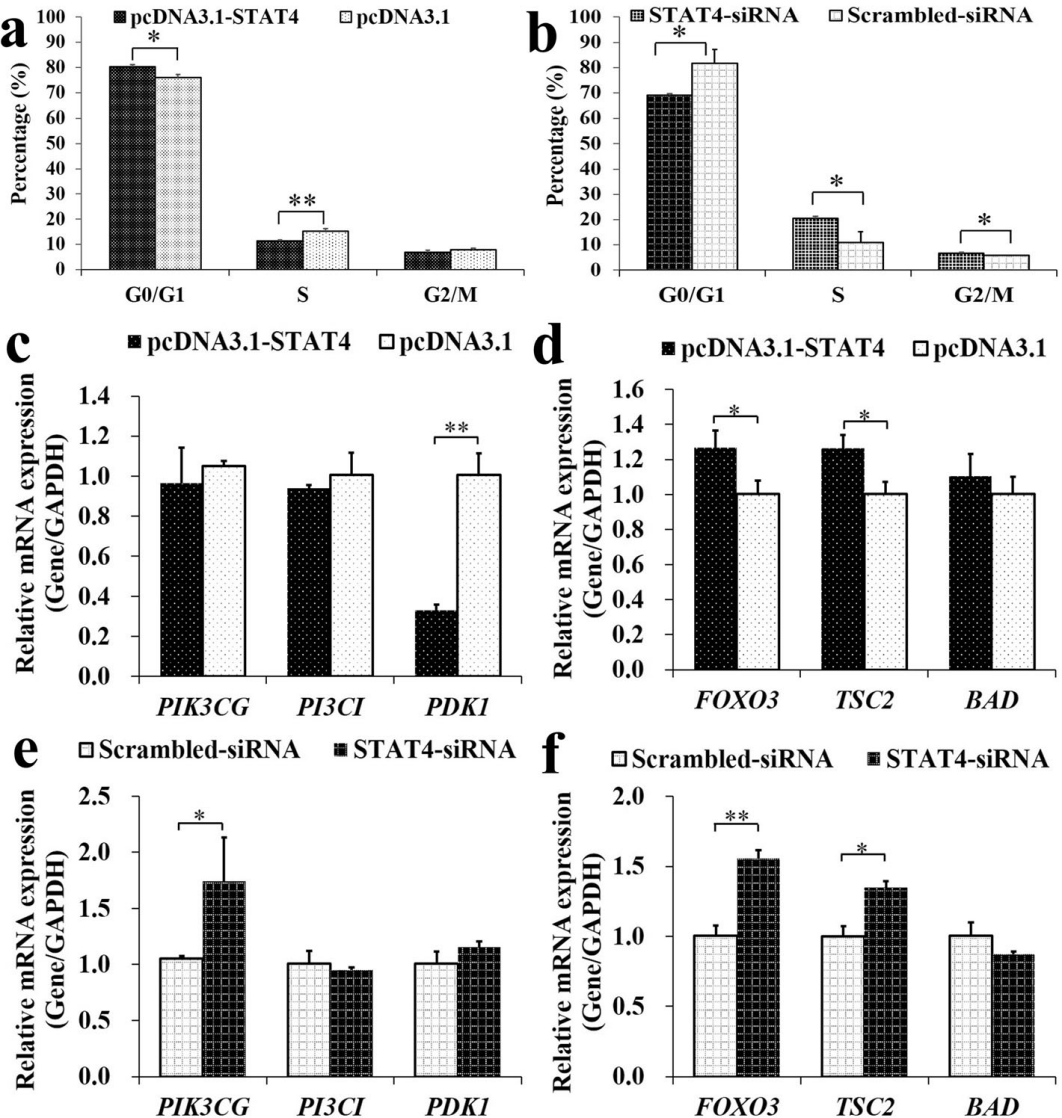
Biological Effects of *STAT4* on cell cycle and PI3K signaling pathway in GCs. **a** Effects of pcDNA3.1-STAT4 on the cell cycle stage; **b** Effects of STAT4-siRNA on the cell cycle stage; **c** Relative mRNA expressions of *PIK3CG, PI3C1,* and *PDK1* after the treatment by pcDNA3.1-KISS1; **d** Relative mRNA expressions of *FOXO3, TSC2,* and *BAD* after the treatment by pcDNA3.1-KISS1; **e** Effects of KISS1-siRNA on the relative mRNA levels of *PIK3CG, PI3C1,* and *PDK1*; **f** Effects of KISS1-siRNA on the relative mRNA levels of *FOXO3, TSC2,* and *BAD*. ** indicates *P* < 0.01; * indicates *P* < 0.05. Data were represented as means ± SD

To further identify the biological function of *STAT4* on cell cycle and cell apoptosis, pcDNA3.1-STAT4 or STAT4-siRNA was transfected into porcine GCs. After the analysis of cell cycle, the pcDNA3.1-STAT4 was observed to significantly upregulate the percentage of cells in the stage of G0/G1 (Fig. 3a) and downregulate the percentage of cells in the stage of S, but the STAT4-siRNA was found to significantly downregulate the percentage of cells in the stage G0/G1 and upregulate the percentage of cells in the stage of S (Fig. 3b).

**Fig. 4:**
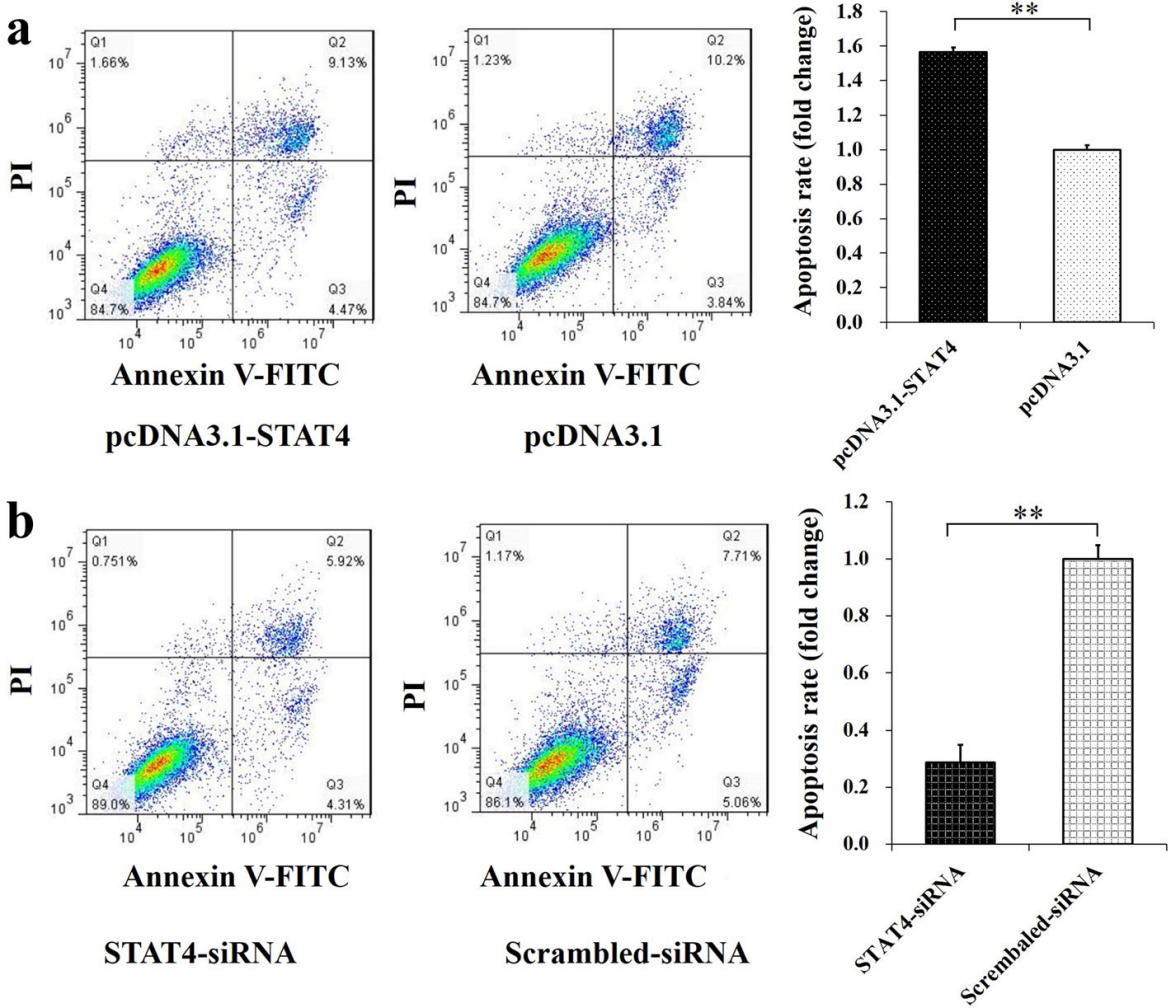
*STAT4* promotes the apoptosis of GCs. The pcDNA3.1-STAT4 decreased cell apoptosis rate (**a**), but the STAT4-siRNA increased cell apoptosis rate of GCs (**b**); The cell apoptosis was assessed by Annexin V-FITV/PI. The cells in lower right quadrant were annexin-positive/PI-negative early apoptotic cells. The cells in the upper right quadrant were annexin-positive/PI-positive late apoptotic cells. Compared to the blank group, the fold change of the percentage of cells undergoing early and late apoptosis were presented in the barplot. ** indicates *P* < 0.01. Data were represented as means ± SD

Furthermore, the pcDNA3.1-STAT4 was found to downregulate the mRNA of *PDK1* (Fig. 3c) and increased the mRNA levels of *FOXO3* and *TSC2* (Fig. 3d) but did not show significant effects on the mRNA levels of *PIK3CG*, *PI3C1*, and *BAD* (Fig. 3c,d). Besides, the STAT4-siRNA markedly upregulated the mRNA levels of *PIK3CG*, *FOXO3,* and *TSC2* (Fig. 3e,f), but did not show significant effects on the mRNA levels of *PI3C1*, *PDK1*, and *BAD* (Fig. 3e,f). Moreover, the pcDNA3.1-STAT4 was observed to significantly promote the cellular apoptosis (Fig. 4a), and the STAT4-siRNA significantly inhibited the apoptosis in porcine GCs (Fig. 4b). These results suggested that STAT4 might disturb the cell cycle and promote cell apoptosis through PI3K signaling pathway in porcine GCs.

### STAT4 inhibits the synthesis of E2 in porcine GCs

**Fig. 5:**
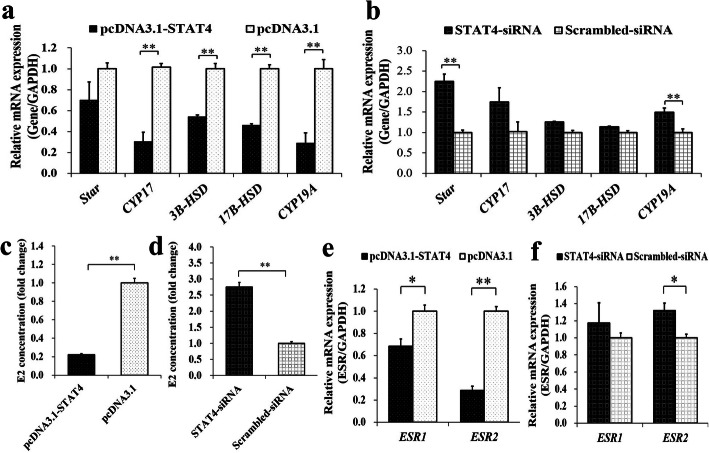
*STAT4* inhibits the synthesis of E2 in porcine GCs. Effects of pcDNA3.1-STAT4 (**a**) and STAT4-siRNA (**b**) on the relative mRNA levels of *Star*, *CYP17*, *3B-HSD*, *17B-HSD*, and *CYP19A*; **c** Concentrations of E2 was depressed by pcDNA3.1-STAT4; **d** Concentrations of E2 was stimulated by STAT4-siRNA; **e** Effects of pcDNA3.1-STAT4 on the relative mRNA levels of *ESR1* and *ESR2*; **f** Effects of STAT4-siRNA on the relative mRNA levels of *ESR1* and *ESR2*. ** indicates *P* < 0.01; * indicates *P* < 0.05. Data were represented as means ± SD

To investigate the biological effect of *STAT4* on the synthesis of E2 in porcine GCs, the pcDNA3.1-STAT4 or STAT4-siRNA was transfected into porcine GCs (Fig. 5). We found that the overexpression of *STAT4* significantly increased the mRNA levels of *CYP17*, *3B-HSD*, *17B-HSD* and *CYP19A* (Fig. 5a), and STAT4-siRNA significantly downregulated the mRNA levels of *Star* and *CYP19A* (Fig. 5b). Furthermore, the pcDNA3.1-STAT4 (Fig. 5c) or STAT4-siRNA (Fig. 5d) significantly decreased or increased the concentration of E2 in porcine GCs, respectively. Additionally, the pcDNA3.1-STAT4 significantly down-regulated the mRNA levels of *ESR1* and *ESR2* (Fig. 5e), and the STAT4-siRNA appeared to increase the mRNA expressions of *ESR1* and *ESR2* (Fig. 5f).

### STAT4 suppresses the effects of KISS1 on cell apoptosis and synthesis of E2 in GCs

**Fig. 6:**
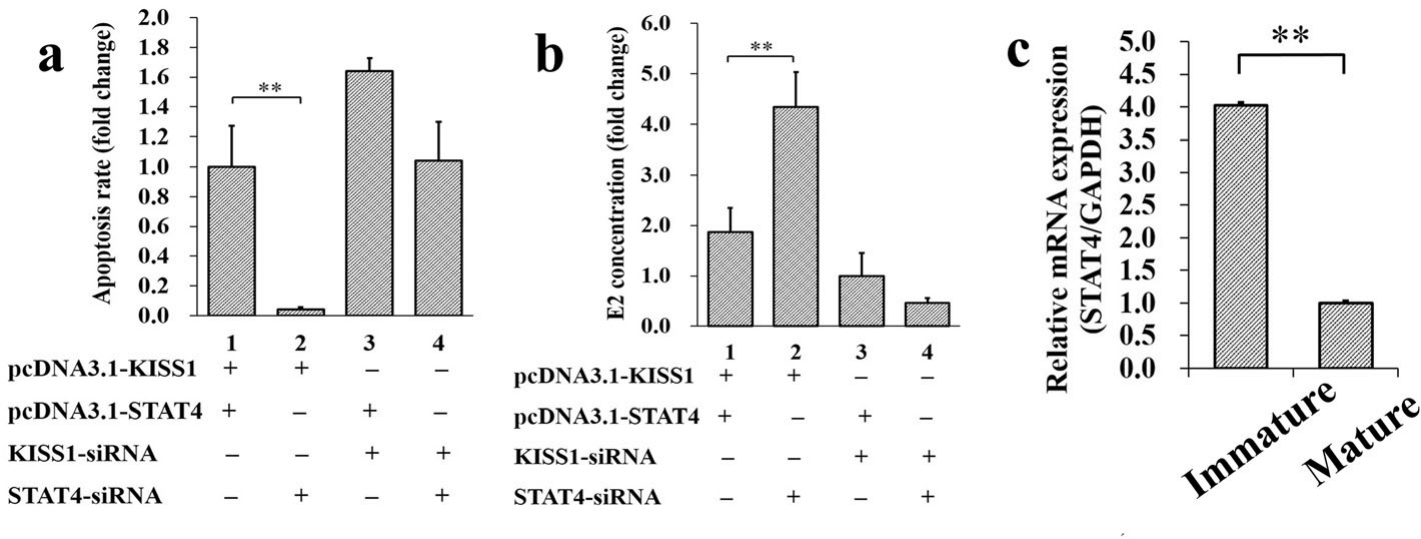
*STAT4* suppresses the effects of *KISS1* on cell apoptosis and synthesis of E2 in GCs. **a** *STAT4* repressed the anti-apoptotic effect of *KISS1* in ovarian GCs; **b** *STAT4* suppressed the synthetic capacity of E2 of *KISS1* in ovarian GCs; **c** Changes of *STAT4* mRNA expression during follicular maturation. ** indicates *P* < 0.01; * indicates *P* < 0.05. Data were represented as means ± SD

To further characterize the biological effect of *STAT4* on the cellular functions of *KISS1*, the pcDNA3.1-STAT4, STAT4-siRNA, pcDNA3.1-KISS1, and KISS1-siRNA were transfected into porcine GCs (Fig. 6). We found that the fold change of group 1 (pcDNA3.1-STAT4 + pcDNA3.1-KISS1) was significantly higher than group 2 (STAT4-siRNA + pcDNA3.1-KISS1) (Fig. 6a) for the cell apoptosis rate, and the synthetic capacity of E2 of group 1 (pcDNA3.1-STAT4 + pcDNA3.1-KISS1) was significantly lower than group 2 (STAT4-siRNA + pcDNA3.1-KISS1) (Fig. 6b). These results indicated that *STAT4* might repress the anti-apoptotic effect and the synthetic capacity of E2 of *KISS1* in ovarian GCs. Moreover, the mRNA level of *STAT4* was observed to significantly decrease from immature to mature follicles (Fig. 6c), which was inversed with that of *KISS1* during the follicular maturation [6].

## Discussion

A series of studies have recently recommended that the *KISS1* might get involved in folliculogensis and controlling the maturation of follicles in mammals [8, 11, 42]. In humans, the level of Kisspeptin is higher in polycystic ovarian syndrome patients than control group, suggesting that the Kisspeptin may be a potential biomarker for the diagnosis of polycystic ovarian syndrome [43]. It is widely known that E2 is mainly synthetized and produced in GCs [44, 45], and the survival and proliferation of GCs directly stimulate the synthesis of E2 [46, 47] and facilitate the follicular maturation [48]. The abnormal apoptosis of GCs appears to provoke the emergence of ovarian endometriosis [49, 50]. Compared with other ovarian cells, the mRNA level of *KISS1* significantly expressed higher in the GCs [11]. The lower expression of *KISS1* in GCs of humans may cause the abnormal follicle development [5]. Moreover, we previously find that *KISS1* stimulates the synthesis of E2 and suppresses cell cycle entry as well as cell apoptosis in porcine GCs [6], while the expression level of *KISS1* mRNA significantly increased from immature to mature follicles [6]. These results indicate that the *KISS1* might regulate the apoptosis of GCs and the synthesis of E2 to promote the follicular development in mammals.

Although the mRNA level of *STAT4* increased along with the concentration of pcDNA3.1-STAT4 at 50 ng, 100 ng, 200 ng and 500 ng, as shown in Fig. 1a, 200 ng of pcDNA3.1-STAT4 was finally selected for further using on the considerations for cellular tolerance, and 200 ng of pcDNA3.1-STAT4 was at the similar concentration level with 50 nM of siRNAs, which was suggested by the providers and instructions. In this study, there were four putative binding sites of STAT4 at the promoter of *KISS1* (Fig. 2a). We found that the deletion of the fourth putative binding site (− 305/− 295) dramatically increased the relative luciferase activity of P5, compared to P0, P1, P2, P3, and P4 (Fig. 2b), and the ChIP was further applied to confirm that STAT4 bound at − 305/− 295 of *KISS1* (Fig. 2c). Besides, the overexpression of *STAT4* significantly decreased the mRNA (Fig. 1b) and protein levels (Fig. 1c) of *KISS1*, and interfering with the expression of *STAT4* significantly increased the mRNA (Fig. 1e) and protein levels (Fig. 1f) of *KISS1* in porcine GCs. Moreover, the mRNA levels of *STAT4* (Fig. 6c) were observed to inverse with that of *KISS1* [6] from immature to mature follicles. These observations suggested that the STAT4 might directly target at − 305/− 295 of *KISS1* to negatively control the transcription of *KISS1* in porcine ovarian GCs.

Accumulating evidence implicates the critical role of PI3K signaling pathway in the survival and apoptosis of ovarian GCs in mammals [32, 33], and the PI3K signaling pathway is suggested to be highly associated with the progression from endometriosis to ovarian cancer [51]. Previous studies recommend that PDK1-deficient in oocytes causes the depletions of the majority of primordial follicles around the first estrus [52]. The DNA structure variation of *PIK3CG* may strike the occurrence of ovarian cancer [53]. In early adulthood of mice, the *TSC2*-deficient in oocytes depletes follicles and results in premature ovarian failure [54]. In mice, the *FOXO3*-deficient in GCs inhibits the ovarian follicular growth [55]. The *PIK3C1* gene, which encodes an isoform of the catalytic subunit of PI3K, is suggested to regulate the follicle survival [32] and involve in the cellular proliferation and apoptosis of ovarian cancer in mammals [56]. The *BAD* gene, known as BCL2 associated agonist of cell death, is found to regulate the apoptosis of ovarian GCs to reduce progesterone levels in sheep [57]. In this study, herein, the effects of STAT4 on the expressions of *PIK3CG*, *PIK3C1*, *PDK1*, *FOXO3*, *TSC2* and *BAD* from PI3K signaling pathway were determined (Fig. 3c-f). We found that the pcDNA3.1-STAT4 down-regulated the mRNA of *PDK1* (Fig. 3c) and up-regulated the mRNA level of *FOXO3* and *TSC2* (Fig. 3d), and the STAT4-siRNA significantly increase the mRNA expressions of *PIK3CG*, *FOXO3*, and *TSC2* (Fig. 3e,f). Additionally, the analysis of Annexin V-FITC flow cytometry showed that the pcDNA3.1-STAT4 promoted the apoptosis of GCs (Fig. 4a), and the STAT4-siRNA inhibited the apoptosis of GCs (Fig. 4b). Moreover, the pcDNA3.1-STAT4 and STAT4-siRNA were found to increase and decrease the number of GCs at phase of G0/G1 in the cell cycle, respectively (Fig. 3a,b). These results are in according to the study that shSTAT4 lentivirus transduces more cells in G0/G1 phase and inhibits the proliferation of vascular smooth muscle [58]. These findings indicated that the *STAT4* involved in PI3K signaling pathway to suppress cell cycle entry and promote cell apoptosis in porcine GCs.

The expression of *Star* gene, which promotes the conversion of cholesterol into pregnenolone, is enhanced by androgen in rat GCs [59], and the *KISS1* is likely to trigger the expression of *Star* in the granulosa lutein cells of humans [60]. The *CYP17* gene encodes one member of the cytochrome P450 enzymes that produce androgens and estrogens, as well as impacting follicular development [61]. The *3B-HSD* gene encodes the enzyme that catalyzes the oxidative conversion and regulates the production of progesterone and E2 in GCs of cattle [62]. In women, the variants in *CYP19A* gene yielded fewer mature follicles [63]. The *17B-HSD* converts the estrone to more active estrogens, and the disorders of *17B-HSD*, estrogen receptors and estrogens cause the prevalence of the ovarian endometriosis [64]. Therefore, in this study, the impacts of STAT4 on several genes regulated the synthesis of E2 were investigated (Fig. 5), including *Star*, *CYP17*, *3B-HSD*, *17B-HSD* and *CYP19A* as well as the estrogen receptor *ESR1* and *ESR2*. We found the pcDNA3.1-STAT4 markedly decreased the mRNA expressions of *CYP17* (Fig. 5a), *3B-HSD* (Fig. 5a), *17B-HSD* (Fig. 5a), *CYP19A* (Fig. 5a), *ESR1* (Fig. 5e), and *ESR2* (Fig. 5e), and decreased concentration of E2 in porcine GCs (Fig. 5c). Whereas the STAT4-siRNA significantly upregulated the mRNA expressions of *Star* and *CYP19A* (Fig. 5b), up-regulated the mRNA levels of *ESR1* and *ESR2* (Fig. 5f) as well as the concentration of E2 in porcine GCs (Fig. 5d). These observations suggested that the STAT4 might suppress the synthesis of E2 in ovarian GCs. Moreover, the STAT4-siRNA could significantly decrease the cell apoptosis rate of pcDNA3.1-KISS1 and significantly increase the concentration of E2 of pcDNA3.1-KISS1, compared to that of the pcDNA3.1-STAT4 (Fig. 6a,b). Moreover, the mRNA level of *STAT4* was observed to significantly decrease from immature to mature follicles (Fig. 6c), which was inversed with that of *KISS1* during the follicular maturation [6]. These results indicated that the STAT4 might repress the anti-apoptotic effect and the E2 synthetic capacity of *KISS1* in GCs to inhibit the follicular development in pigs.

## Conclusions

Taken together, the STAT4 might directly target at − 305/− 295 region of *KISS1* to negatively regulate the transcription and biological of *KISS1*, involve in PI3K signaling pathway to promote the cell apoptosis, and participate in estrogen synthesis signaling pathway to suppress the synthesis of E2 in GCs to arrest the follicular development. These proposed works would contribute to providing new biological insight for further investigation on *STAT4* and *KISS1* in the follicular development of mammals.

## Data Availability

The dataset supporting the conclusions of this article are included within the article.

## Abbreviations

GCs: Granulosa cells
KISS1: Kiss-1 metastasis-suppressor gene
STAT: Signal transducer and activator of transcription
E2: Estradiol-17β
PI3K: Phosphatidylinositol 3-OH-kinase
SiRNA: Small interfering RNA
ChIP: Chromatin immunoprecipitation
PBS: Phosphate-buffered saline
SD: Standard deviation
